# Apoplexy, Complicating Cirrhosis of the Kidneys—Embolism of the Right Cerebral Artery—Obscure Abdominal Tumor

**Published:** 1883-10

**Authors:** James H. Hutchinson

**Affiliations:** One of the Attending Physicians to the Hospital, Physician to the Children’s Hospital, Etc.


### BUFFALO

### Medical and Surgical Journal.

Vol. XXIII. OCTOBER, 1883. No. 3.

### Original Communications.

### Apoplexy, Complicating Cirrhosis of the Kidneys—Embolism of the Right Cerebral Artery—Obscure Abdominal Tumor.

A CLINICAL LECTURE DELIVERED AT THE PENNSYLVANIA HOSPITAL.

By James H. Hutchinson, M. D.,
One of the Attending Physicians to the Hospital, Physician to the Children's Hospital, Etc.

Reported by William H. Morrison, M. D.

Gentlemen—I shall begin the lecture this morning by showing you the specimens removed from the body of a man who was admitted to the hospital on Saturday afternoon last, and who died in the course of twenty-four hours. He was suddenly taken on the street with paralysis and unconsciousness and was brought to the hospital, where an examination of the urine showed that it contained albumen. He was unconscious; there was no marked paralysis; there was no stertorous respiration, and there was albumen in the urine. It therefore seemed a sufficient explanation of the case to suppose that the man was suffering from uræmic coma. The examination of the kidneys after death revealed marked alteration of their structure. I here show you the kidneys. There is a great diminution of the cortical tissue, while the pyramidal tissue is not particularly affected. In some places the cortical substance is not more than a line in thickness. The surface of the kidney is contracted and presents the appearance which is indicative of the change known as cirrhosis. Looking at the cut section of the organ, you see that the kidney is filled with a large number of little cysts. These are due to the fact that the contraction of the intercellular tissue obstructs the tubules, and their contents, prevented from being excreted, undergo changes and cysts are developed. In the kidney from the other side, the capsule has not been removed and you observe that as I try to take it away I tear off some of the renal tissue. This granular appearance of the organ is due to the contraction of the intercellular tissue which compresses the tubules and greatly interferes with the functions of the kidney.

A further examination of the body revealed that the man was also the subject of apoplexy, a clot being found in the lateral ventricle of the right side. I here show you the clot. As I have said there was in this case no stertor of the respiration, which we should expect to find in a case of apoplexy. It is possible that the hemorrhage came on later. You are all probably aware that in disease of the kidney, especially of the cirrhotic form, there is always more or less alteration in the structure of the arteries, the minute vessels suffering more than the larger ones. They become more friable and more liable to give way, hence apoplexy is by no means an uncommon complication of kidney disease. The absence of stertor, marked paralysis and elevation of temperature and the presence of albumen in the urine led us to diagnose uræmic coma.

There are, I think, some useful lessons to be drawn from this case. This man was going about and therefore apparently not very ill. It is not at all rare for an uræmic seizure to occur suddenly, and it frequently happens that disease of the kidneys exists in those in whom it has never been suspected. I know of no other disease which is so apt to come on insidiously and finally culminate in death so rapidly as Bright's disease of the kidneys.

I well recollect the case of a woman with a form of skin disease which was in the hospital some years ago. The affection was indeed so slight that it hardly arrested my attention. After being in the house for several days, she was seized with intense dyspnœa, which was so extreme that I could not satisfactorily account for it. Within twenty-four or thirty-six hours she died. At the post mortem, I found both kidneys much reduced in size; one of them did not weigh more than seven drachms. There was no history of kidney trouble and there were no symptoms of such an affection, and the disease for which she was admitted was so slight that the customary examination of the urine was omitted. If this had been attended to, albumen would probably have been found; not certainly, however, for in many instances of cirrhosis of the kidney, albumen is absent from the urine.

Again turning to the specimens, I call attention to a slight enlargement of the heart, which is not an infrequent complication of cirrhotic disease of the kidney. You can, I think, readily understand why there should, under such circumstances, be hypertrophy of the heart. The minute arteries have undergone changes which impose an additional work upon the heart. These arteries, by their contraction, aid in propelling the blood. If they are altered in structure so as to be unable to assist in the circulation of the blood, additional labor is thrown on the heart, which induces hypertrophy. As a secondary effect of the condition of the smaller arteries, we frequently have valvular disease.

The lungs are very dark in color, which may be a result of his occupation. There is no disease of these organs.

In regard to the treatment of such a case, there is very little to be done. Even if we had suspected the existence of apoplexy, it is not at all likely that we should have bled. The only thing to be done, under such circumstances, is to try to sustain life. There is no indication for medicine. Sometimes in uræmic coma, diuretics and hot baths are employed, but in a person ill as this man was, little could be hoped for from the use of remedies.

### EMBOLISM OF THE RIGHT CEREBRAL ARTERY.

I shall next bring before you a patient who presents some points of interest. He has been in the hospital before, and left a short time ago, but he has recently returned, with a new set of symptoms superadded to those which he before presented. This is his history: D. F., age 21; born in Ireland; married; a waiter by occupation. He was admitted on the 19th of the present month (February). The family history is good. He was always healthy until four years ago, when he had an attack of inflammatory rheumatism, lasting for six weeks. This was soon followed by pain in the chest, palpitation of the heart and shortness of breath on exertion. These symptoms continued for two months, and he then noticed that the face began to swell. Shortly afterwards the feet, hands and abdomen also became swollen. He was admitted to the hospital under the care of my colleague, January 8, 1883, with general anasarca and valvular disease of the heart. On the 3d of the present month he left the house a good deal improved. He remained quite well until night before last, February 17th, when he found himself lying on the floor, having fallen out of bed, with the left arm and leg paralyzed, and suffering from severe headache. He denies having had any venereal disease, and there is no history of syphilis. On admission, "the patient was found to be well nourished, has headache, seems dull and restless, the appetite is poor and the bowels constipated. The tongue is slightly coated. There is some œdema of the face, but no swelling of the hands or feet. The left arm and leg are completely paralyzed. There is slight ptosis of the right eyelid. The left side of the face is also paralyzed, and the tongue is protruded to the left side.

Sensation is a good deal impaired in the paralyzed parts." There is also a note in regard to the cardiac disease, to which I shall not at present refer.

We have here a man who, after leaving the hospital, remained guite well until a day or two ago, when he found himself lying on the floor with paralysis of the left side. As he lies here upon his back, you can see that there is paralysis of the left side of the face. When he attempts to protrude the tongue, it is directed toward the left side. There does not seem to be any inability to close the eyelids, but there seems to be a slight ptosis on the right side, *i. e.*, some paralysis of the third nerve of the right side. I am, however, not perfectly sure that such is the case, but the eyelid certainly droops.

Having a paralysis occur in this way in a robust young man, we must, I think, conclude that it is due to one of two causes: either to embolism or to syphilitic disease of the brain. It may, of course, be dependent upon some other disease of the brain, but this is unlikely. The question is to decide between these two conditions. There is the history of early cardiac disease, which was clearly recognized, and there is no admission on the part of the patient of any venereal disease, and there is nothing in the history or in the present symptoms which would indicate any syphilitic trouble. I think, therefore, that there is no doubt as to the nature of the disease. This is a case of embolism of the brain, and the third which you have seen within a few weeks.

So much has been said about the mechanism of this affection that it may seem tiresome to again refer to the subject, but as you are likely to meet with the disease quite frequently, it is important that it should be thoroughly understood. In the other two cases which you have seen, there was mitral constriction and mitral regurgitation. In this boy, in addition to mitral regurgitation, there is also disease of the aortic valves. I think that you can readily understand that if there is an impediment at the mitral orifice, embolism may very easily occur.

In such a condition it is common to have vegetations; and more than this, coagulation of blood in the left auricle is very apt to take place. There is an obstruction to the passage of the blood to the left ventricle, and consequent coagulation of blood in the auricle. I have not unfrequently met with this at post mortem examinations. This clot may become to a certain extent broken up, and a portion be carried into the circulation until it reaches an artery whose calibre it partially or entirely obliterates. It is not necessary that it should completely obstruct the vessel, but only that the embolus should be arrested, for the subsequent coagulation effectually closes the artery.

In this boy the symptoms appear to have come on suddenly and during sleep. This is not at all uncommon. One would be more apt to expect that the clot or vegetation would be detached during active exercise, but this is by no means always the case.

There is one point about this case, and the same thing was true of one of the other patients, and that is, that the paralysis affects the left side. In the greater number of cases, the right side of the body is the part affected in embolism. The reason of this is that the course of the left carotid is a little more direct than that of the right, and consequently the embolism is more apt to pass into the former vessel and obstruct some of its branches, thus giving rise to paralysis of the right side. There is, however, no reason why the right carotid should not occasionally be the seat of embolism.

In regard to the prognosis, this depends to a certain extent upon the size of the artery obliterated. If it is a large one, there is less probability of an entire return of function than if the vessel is a small one; in the latter case, the collateral circulation may be sufficient to take the place of the occluded artery. In both of the other patients who have been before you, there has been great improvement.

We unfortunately do not possess any remedy which is capable of dissolving the clot in the artery. The alkalies seem to be indicated, and if it were safe to give them is such doses as would produce a solution of the clot, I think that we should probably succeed. They, however, produce such debility that they would do more harm than good. The treatment is expectant. If there is œdema, or other symptom indicating cardiac trouble, we should give digitalis or diuretics, as Basham's mixture. In the present case, there is no particular indication, and I do not see that we can do much more than wait. Iodide of potassium might be prescribed, but it is not likely to have much effect upon the clot.

### OBSCURE ABDOMINAL TUMOR.

This case presents some points of interest in reference to the diagnosis. This is his history: "M. W.; age 29; born in Ireland; married; by occupation, a miner; he was admitted February 19, 1883; the family history is good; he had scarlet fever at the age of nine years, and jaundice fifteen years ago; since then he has had good health until six months ago, when he noticed a pain in the stomach which has troubled him ever since; his appetite has failed and he has lost flesh and strength; four weeks ago, while vomiting, he noticed, for the first time, a tumor in the upper part of the abdomen; he has had but two attacks of vomiting, one occurring four and the other two weeks ago; on these occasions the sickness appeared to be due to medicine that he had taken; he has never vomited blood; the pain is sharp and cramp-like, but not constant, being worse when the stomach is empty; he denies any venereal disease and there is no history of syphilis; he has six children living and healthy; he never suffered from malarial poisoning and has never received an injury to the stomach; on admission, he was found to be anæmic, but well nourished; the bowels regular; appetite poor; tongue slightly coated, but moist."

We have had quite a number of cases of abdominal tumors this year, and such cases always present considerable difficulty in the diagnosis. One would suppose that the walls of the abdomen being so thin and apparently presenting no interference with the examination of a mass within the abdomen, that the diagnosis of abdominal tumors would be among the easiest that we are called upon to make. The fact is that they are among the most difficult. In tumors of the brain we are often able to exactly locate the growth during life. You will recollect that in the case which I showed you a short time ago, the diagnosis of a tumor at a certain portion of the base of the brain which I had made during life, was verified by the post mortem examination. So in diseases of the lung, there is comparatively little difficulty in making out the seat of the disease and also its nature. But in regard to abdominal tumors, I have been at times puzzled to determine their exact nature.

We have here a patient with an abdominal tumor, and I must say that I have not been able to determine its exact seat and nature. I have seen him but once, and as he will probably leave the house in a short time, I have brought him before you, for it is important that you should see not only those cases in which we have been successful in arriving at the diagnosis, but also those in which we have failed.

You can readily see a prominence in the left hypochondriac region. If it remained in this position all the time, we should be apt to conclude that it was due to enlargement of the spleen. It is, however, not always in that position. When first discovered, it was lower down and in the median line. We are able to push it about, and not only so, but movement of the patient's body also causes its displacement. When he turns on the right side, you see that the mass falls to that side. When he sits up, the tumor descends. When I first saw him, the mass was still lower than it is at present. It therefore changes its position very considerably. You will recollect that in the early part of the course I showed you an old colored woman with a tumor which was very movable, but not so movable as is this one. The diagnosis in this case also gave considerable trouble. The disease occurred in a woman over eighty years of age; there had been no symptoms of gastric trouble, yet after death he found a cancerous tumor of the pylorus.

In the present case, it seems almost impossible that this mass should be connected with the stomach. The patient has had no vomiting except on two occasions within the past six weeks, and it was then apparently due to something that had been taken. This is hardly sufficient to make out the diagnosis of cancer of the stomach. This affection occasionally occurs without producing sickness of the stomach, but this is rare. Cancer is also less frequent before middle life than it is after that period.

From the great degree of movement, it seems to be more probable that this is an omental tumor.

You might ask if this mass was not a floating kidney. From the degree of movement and from the fact that the man has been losing flesh and strength, this seems improbable. We have therefore to do with a tumor the exact nature and seat of which we can not exactly determine.

In treating such a case we should do all that is possible to build the patient up. Tonics and good nutrient food should be given. In the way of medicine I do not know of anything more likely to meet the indications better than the iodide of iron. I shall therefore order the syrup of the iodide of iron in half teaspoonful doses, three times a day. If there should be any strumous condition giving rise to this tumor, this would meet the indications.

At the present time it is imposible to speak more positively of the prognosis than we have of the diagnosis. This question can only be settled by time. If the patient should remain in the hospital and give me a further opportunity of studying his case, I shall again bring him before you.

